# The Gut Microbiota–Tryptophan–Brain Axis in Autism Spectrum Disorder: A New Frontier for Probiotic Intervention

**DOI:** 10.3390/microorganisms14020312

**Published:** 2026-01-29

**Authors:** Yi Cheng, Liangyu Zhang, Yalin Li, Chunru Zheng, Teng Ma, Zhihong Sun

**Affiliations:** 1 Key Laboratory of Dairy Biotechnology and Engineering, Ministry of Education, Inner Mongolia Agricultural University, Hohhot 010018, China; hhht_bb@163.com (Y.C.); lyzhang9910@163.com (L.Z.); lylzbd0613@163.com (Y.L.); 13948911793@163.com (C.Z.); 18447054019@163.com (T.M.); 2 Key Laboratory of Dairy Products Processing, Ministry of Agriculture and Rural Affairs, Inner Mongolia Agricultural University, Hohhot 010018, China; 3 Inner Mongolia Key Laboratory of Dairy Biotechnology and Engineering, Inner Mongolia Agricultural University, Hohhot 010018, China

**Keywords:** tryptophan metabolism, gut microbiota, autism spectrum disorder, gut–brain axis, probiotics

## Abstract

Tryptophan (Trp) metabolism is involved in regulating various physiological and pathological processes, including neurological function, immune response, and gut homeostasis. This article focuses on autism spectrum disorder (ASD) and explores its relationship with abnormalities in the gut microbiota–Trp–brain axis. Studies have shown that ASD patients exhibit Trp metabolism disorders, with gut microbiota dysbiosis inducing systemic inflammation, activating indoleamine 2,3-dioxygenase 1 (IDO1), and promoting increased Trp entry into the kynurenine pathway (KP). This leads to a series of pathological changes, including the production of neurotoxic substances, serotonin system disorders, and impaired intestinal barrier function, which in turn exacerbate ASD symptoms through the gut–brain axis. Furthermore, based on preclinical and clinical studies, we have summarized that specific probiotic strains (such as *Lactobacillus* and *Bifidobacterium*) can alleviate the clinical manifestations of ASD by regulating the gut microbiota–Trp metabolic axis, improving immune responses, and enhancing intestinal barrier function. We emphasize that current probiotic interventions still face challenges such as insufficient long-term safety assessments and unclear molecular mechanisms. Future research should combine multi-omics technologies and multi-modal approaches to promote the development of personalized and precise intervention strategies. In summary, this review highlights the crucial role of tryptophan metabolism in ASD and the potential of probiotics as a novel adjunctive therapy targeting this metabolic pathway.

## 1. Overview of ASD

ASD encompasses a complex group of neurodevelopmental disorders. The latest edition of the Diagnostic and Statistical Manual of Mental Disorders (DSM) collectively classifies Asperger’s syndrome, autism, childhood disintegrative disorder, and other pervasive developmental disorders not otherwise specified as ASD [1]. ASD is characterized by persistent deficits in social communication and interaction, as well as restricted, repetitive patterns of behavior, interests, or activities [2]. ASD is associated with high levels of anxiety, stress, and isolation in affected families, while the individuals themselves require significant care and experience reduced productivity, placing a heavy economic burden on society and their families [3,4]. Epidemiological surveys show an increasing annual prevalence of autism, a phenomenon attributed to various factors, including an increased true prevalence of ASD, a broader definition of the disorder, changes in diagnostic criteria and screening tools, shifts in research methods, and increased awareness of ASD [5]. According to the latest Global Burden of Disease study released in 2024, as of 2021, 61.8 million individuals worldwide had ASD (1 in 127), and this number continues to rise [6]. The prevalence rate is consistently higher in boys than girls, and there are significant differences between age groups. The highest diagnosis rate is in children aged 0–4 years, with the prevalence decreasing with age; however, the diagnosis rate in people aged 60 and older has been increasing in recent years.

ASD is a neurobiological disorder influenced by both genetic and environmental factors, affecting brain development, but the ultimate pathogenic mechanism remains unclear. Current main treatment strategies include medication and dietary adjustments, with gluten-free diets, casein-free diets, and ketogenic diets receiving widespread attention due to their demonstrated clinical benefits [7]. However, current research has limitations. Low patient adherence, the heavy economic burden of treatment, and insufficient consideration of individual differences all limit the generalizability and reliability of existing research results. Consequently, the development of effective therapeutic strategies has become a dominant theme in the field. In addition to neurological symptoms, ASD patients frequently experience gastrointestinal symptoms, and a growing body of research has proposed the role of the gut microbiota in both gastrointestinal symptoms and neurodevelopmental dysfunction in ASD patients. The gut microbiota and the central nervous system form a complex bidirectional communication network, the “gut-brain axis”. This network, through various mechanisms including neurotransmission, hormonal regulation, and immune pathways, synergistically regulates brain function and digestive system activity, exerting a significant influence in early development and shaping fundamental neural circuits and cognitive functions [8,9].

Furthermore, some gut microbiota metabolites, as key signaling molecules and chemical mediators, influence the central nervous system through multiple pathways, including neural, immune, and circulatory pathways, directly or indirectly participating in neurodevelopment, neuroinflammation regulation, and maintaining the integrity of the blood–brain barrier. Examples include short-chain fatty acids, Trp metabolites, secondary bile acids, γ-aminobutyric acid (GABA), and dopamine. In this complex interactive system, Trp metabolism is considered a crucial bridge [10]. As an important precursor to neuroactive substances such as 5-HT and kynurenine (Kyn), Trp metabolic pathway directly connects the gut microbiota ecology with central nervous system function, playing a central role in gut–brain communication. Therefore, understanding and regulating tryptophan metabolism is a key breakthrough for future interventions in mental illnesses through diet, probiotics, and other means. This review aims to systematically explore the role of the “gut microbiota-tryptophan metabolism-brain axis” in the development of ASD and to elucidate that probiotics, by regulating this axis, may become a potential adjunctive treatment strategy to improve the core symptoms of ASD.

## 2. Materials and Methods

### 2.1. Search Strategies

A systematic literature search was performed in CNKI, PubMed, Cochrane Library, Web of Science, ScienceDirect, and Medalink by 1 July 2025, using search terms such as (“Autism Spectrum Disorder” OR “ASD” OR “Autism” OR “Autistic Disorder” OR “Asperger’s syndrome” OR “Asperger’s disorder” OR “autistic traits”) AND (“Microbiota” OR “Microbiome” OR “Probiotic” OR “Probiotics”).

### 2.2. Inclusion Criteria and Exclusion Criteria

Inclusion and exclusion criteria were established according to the PICOS framework (Participants, Interventions, Comparisons, Outcomes, Study design).

The inclusion criteria were as follows:

(1) Children and adolescents under 20 years of age diagnosed with ASD, autistic disorder, or Asperger’s syndrome according to generally accepted diagnostic criteria;
(2) Use of probiotics or probiotic preparations as the primary intervention in the experimental group;
(3) No restrictions on control group interventions;
(4) Measurement of autism-related behavioral symptoms using validated scales;
(5) Study designs: randomized controlled trials (RCTs) and crossover trials.

Exclusion criteria for the systematic review:

(1) Participants aged 18 years or older;
(2) Full-text articles unavailable after contacting the authors.

Exclusion criteria for the meta-analysis:

(1) Participants aged 18 years or older;
(2) Full-text articles unavailable after contacting the authors;
(3) Insufficient data for meta-analysis;
(4) Lack of information on the specific probiotic strains used.

### 2.3. Study Selection and Data Extraction

Two researchers independently screened the literature by evaluating titles and abstracts. Studies meeting the criteria underwent full-text review by the same researchers. Data were independently extracted, including basic participant characteristics, sample size, intervention and control details, intervention duration, scores for autism-related behavioral symptoms, and scores for GI symptoms.

## 3. Gut Microbiome Characteristics and Accompanying Pathophysiological Changes in ASD Patients

In the gut, trillions of microorganisms form a complex and dynamic micro-ecosystem called the gut microbiota, which is crucial for gut health and immune function. Numerous studies have shown that ASD patients commonly exhibit dysbiosis compared to healthy individuals, characterized by a significantly increased Firmicutes/Bacteroidetes ratio and altered composition of major bacterial phyla. Strati et al., in their analysis of the gut microbiota of autistic patients, found that at the genus level, the relative abundance of genera such as *Dialister*, *Parabacteroides*, and *Veillonella* was decreased, while the relative abundance of *Collincella*, *Corynebacterium*, *Dorcella*, and *Lactobacillus* was increased [11]. Ding et al. proposed a significant increase in the relative abundance of genera such as *Dorcella*, *Collincella*, and *Clostridium*, while *Bacteroides*, *Faecalibacterium*, *Parasutterella*, and *Paraprevotella* were significantly lower than in healthy children [12]. Overall, individuals with ASD exhibit signs of gut microbiota dysbiosis, with reduced overall diversity and varying abundances of *Bacteroidetes*, *Firmicutess*, *Prevotella*, *Fusobacterium*, *Akkermansia*, and many other microorganisms compared to healthy controls. While the specific changes in bacterial genera vary across studies, they collectively reveal a core fact: the gut microbiota dysbiosis in ASD is not dominated by a single pathogenic microorganism but rather constitutes a systemic imbalance involving the overall structure and diversity of the microbiota. This dysbiosis manifests as alterations in the composition of the microbial community, leading to a disruption in the stability of the micro-ecosystem.

An important link between the gut microbiota and the immune system is that inflammation and immune dysregulation have been shown to be associated with the development and severity of ASD [13]. Due to microbiota dysbiosis, the activated immune system releases chemokines and cytokines such as interleukin-6 (IL-6), interferon-γ (INF-γ), and tumor necrosis factor-α (TNF-α). These mediators can cross the blood–brain barrier, bind to endothelial cells in the brain, and induce an immune response. Studies by Vargas et al. have found significant neuroinflammatory activity in the cerebral cortex and white matter (particularly the cerebellum) of ASD patients, with significantly elevated expression levels of inflammatory factors such as TGF-β1, IL-6, and IL-10 in brain tissue [14]. This neuroinflammatory response, characterized by the persistent activation and proliferation of glial cells, is usually triggered by injury, infection, or disease states [15]. Its development can lead to abnormal brain function, which may exacerbate the core symptoms of ASD. Studies have also shown that persistent immune dysregulation leads to a significant alteration of the Th17/Treg balance in ASD patients. This imbalance also disrupts the integrity of the intestinal barrier. Upregulation of Th17 cell differentiation or development results in excessive signaling activity, leading to elevated levels of inflammatory markers in the brain, such as TNF-α, interferon-γ, IL-2, IL-4, IL-5, and IL-6 [16,17]. These inflammatory markers disrupt the balance between excitatory and inhibitory synaptic transmission, activating microglia and releasing inflammatory mediators like IL-1β and NO [18]. Excessive NO interacts with N-methyl-D-aspartate receptors, exacerbating excitotoxicity and inducing neuroinflammation. On the other hand, brain-derived neurotrophic factor (BDNF) is a key neurotrophic factor supporting neuronal survival and promoting synaptic growth and maturation [19]. Excessive inflammatory mediators downregulate BDNF expression, interfering with synaptic structure and function, leading to neuropsychiatric symptoms such as depression and cognitive impairment, ultimately affecting social and cognitive function.

Another study also reported that children with ASD exhibiting congenital pro-inflammatory responses or impaired T-cell activation showed more severe behavioral problems compared to children with ASD exhibiting non-inflammatory or non-T-cell activation immunological characteristics [20]. Simultaneously, the released pro-inflammatory factors activated IDO1, the rate-limiting enzyme in the step of Trp catabolism to Kyn, driving Trp into the KP and resulting in a significant reduction in Trp available for serotonin synthesis. IDO1 activation is a key node connecting immunity and Trp metabolism, primarily mediating immune regulation through a dual mechanism: on the one hand, Trp depletion activates GCN2 kinase within T cells, triggering an integrated stress response that directly inhibits the proliferation and function of effector T cells and induces their apoptosis; on the other hand, the accumulation of the metabolite Kyn can act as an endogenous ligand to activate the aryl hydrocarbon receptor signaling pathway, driving naive T cells to differentiate into immunosuppressive regulatory T cells [21]. This synergistic effect of substrate depletion and product accumulation ultimately leads to an imbalance and suppression of the immune response. This is also considered one of the key mechanisms by which gut microbiota dysbiosis affects brain function and induces neuropsychiatric symptoms such as depression and anxiety through the IDO1/KP.

**Figure 1:**
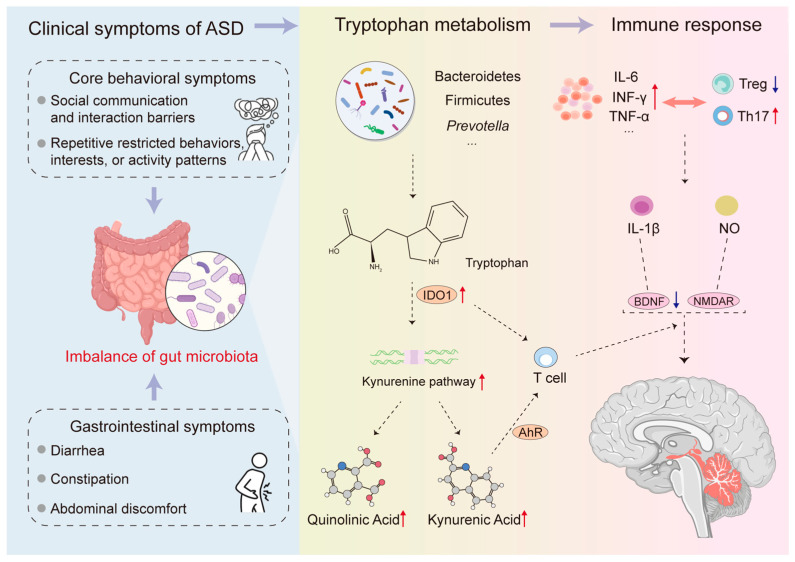
Interaction among Gut microbiota, Trp metabolism, and Immune response. This figure illustrates how gastrointestinal and behavioral symptoms in ASD patients reflect gut microbiota imbalance (dysbiosis), leading to Trp metabolism disorders and consequently systemic immune and neurological dysfunction. Imbalances in key bacterial phyla (e.g., *Bacteroidetes*, *Firmicutes*, *Prevotella*) and upregulation of the host enzyme IDO1 shift Trp metabolism towards the KP. This results in the accumulation of metabolites such as KA and QA. Simultaneously, it exacerbates the immune response, primarily manifested as elevated levels of pro-inflammatory cytokines (IL-6, IFN-γ, TNF-α, IL-1β) and an imbalance in the TH17/Treg cell ratio. Key metabolites can also affect T cell differentiation by acting on AhR. Furthermore, these metabolic changes influence the gut–brain axis by inhibiting BDNF production and modulating NMDAR receptor signaling, as shown in the figure. Abbreviations: IDO1, indoleamine 2,3-dioxygenase-1; KP, kynurenine pathway; KA, Kynurenic Acid; QA, Quinolinic Acid; Treg, regulatory T cell; AhR, aryl hydrocarbon receptor; BDNF, brain-derived neurotrophic factor; NMDAR, N-methyl-D-aspartate receptor.

A growing body of research indicates abnormal metabolite levels in children with autism. A recent analysis of short-chain fatty acids (SCFAs) in the feces of children with ASD found that their valerate levels were twice as high as in healthy children, suggesting that SCFAs present in the diet and those produced by gut microbiota after carbohydrate fermentation may act as environmental triggers for ASD [22]. Valerate levels are also associated with some Lactobacilli in the gut. Another clinical study found lower levels of indole and higher levels of 3-methylindole in fecal samples from children with ASD, both of which are Trp metabolites associated with *Clostridium* species commonly found in patients [23]. Some gut microbiota metabolites also participate in neurotransmitter metabolism, such as the aromatic amino acid derivatives dopamine (DA) and norepinephrine, and the glutamate derivative GABA, which are major mediators of excitatory and inhibitory neurotransmission [24]. Dysregulation of neurotransmitter metabolism is increasingly believed to be related to the pathophysiology behind the cognitive, behavioral, and various neurodevelopmental manifestations observed in ASD, and is regulated by the gut microbiome. This is consistent with previous reports of abnormal levels of neurotransmitters such as 5-HT, DA, GABA, and indole in children with autism, in which the gut microbiota participates in the synthesis and metabolism of neurotransmitters [25,26]. Among the gut microbial metabolites associated with the pathophysiology of ASD, the Trp metabolic pathway has attracted particular attention, with serotonin considered to play a key role in transmission. Clinical data indicate that approximately 30% of ASD patients have elevated levels of 5-HT in their blood, which are associated with dysregulation of symptoms such as mood, appetite, and social interaction. Although these 5-HTs cannot directly cross the blood–brain barrier, they can affect brain function through vagal nerve signaling or by regulating intestinal motility [27,28]. Overall, ASD patients exhibit multi-system dysfunctions, and these abnormalities may play a pathogenic role in a significant proportion of patients, involving multiple aspects such as the gut microbiome and metabolites, immune regulation, and neuroinflammation (Figure 1).

## 4. Trp Metabolism: A Core Bridge Connecting Gut Microbiota and ASD

### 4.1. Trp Metabolism

Trp is an essential amino acid obtained through diet. It plays a crucial role in protein biosynthesis and serves as a precursor for the synthesis of many important bioactive compounds. Trp influences various pathophysiological processes, including metabolism, inflammatory responses, oxidative stress, immune responses, and gut homeostasis. It also participates in regulating circadian rhythms, stress responses, and cognitive function. Trp metabolism in the gastrointestinal tract mainly occurs through three pathways: the KP, the serotonin pathway, and the indole pathway. Specifically: 1. The KP mainly occurs in immune cells and epithelial cells, metabolizing Trp into a series of substances with immune and neurological activity; 2. The serotonin pathway (5-HT) depends on the catalytic action of Trp hydroxylase 1 (TpH1) in enterochromaffin cells to generate 5-HT, which plays a key role in regulating intestinal motility, mood, and cognition; 3. Gut microbiota directly metabolizes Trp into indole and its derivatives. These metabolites are mainly composed of aryl hydrocarbon receptors, helping to maintain the integrity of the intestinal barrier and maintain gut homeostasis [29].

The KP, first described in 1947 as the primary pathway for Trp metabolism, occurs mainly in the liver [30]. During this pathway, over 95% of tryptophan is degraded into various bioactive compounds. Tryptophan-2,3-dioxygenase (TDO), IDO1, and IDO2 are key rate-limiting enzymes in the KP. The cleavage product Kyn is located at a branch point in this pathway; one branch produces the neuroprotective kynurenic acid (KA), while the other branch produces the neurotoxic quinolinic acid (QA). QA is associated with various brain diseases, including Alzheimer’s disease, depression, and schizophrenia [31]. QA is converted to nicotinamide adenine dinucleotide (NAD+) under the catalysis of QA phosphoribosyltransferase and NAD synthase. NAD+ is an important coenzyme in energy metabolism and also plays a crucial role in cell division and mitochondrial function. 5-HT, also known as serotonin, is a monoamine neurotransmitter in the human central nervous system and is an important gastrointestinal signaling molecule that transmits signals from the gut to intrinsic and extrinsic neurons [32]. 5-HT synthesis occurs in the gut and brain [33]. Peripheral 5-HT is mainly synthesized by TPH1 in enterochromaffin cells, which cannot cross the blood–brain barrier and cannot regulate central nervous system function. It mainly affects intestinal peristalsis and motility, secretion, vasodilation, and nutrient absorption. Raphe neurons selectively express TPH2, promoting 5-HT production in the central nervous system [34]. By regulating neuronal excitability and inhibition, TPH2 plays a crucial role in signal transmission between neurons. A very small amount of Trp is converted into indole and its derivatives under the catalysis of tryptophanase, such as indoleacrylic acid, indole-3-acetic acid (IAA), and indole-3-propionic acid (IPA). Many indoles and their derivatives are ligands for aryl hydrocarbon receptors (AhRs) [35]. Activated AhRs participate in immune regulation and various cellular processes, promoting the release of some cytokines, including IL-6, IL-17, and IL-22, thereby regulating immune responses and maintaining intestinal homeostasis. The Trp metabolic pathway is considered a universal therapeutic target for a variety of diseases, including tumors, neurodegenerative diseases, autoimmune diseases, and metabolic diseases, and its metabolic balance is of great significance for maintaining human health (Table 1).

### 4.2. The Link Between Trp Metabolism Imbalance and ASD

Several studies on the humoral metabolomics of children with ASD have shown abnormalities in Trp metabolism in both blood and urine [36]. Several significantly altered metabolic pathways in urine—Trp, purine, and gut microbiota metabolism—are also abnormal in animal models of ASD. The most common biochemical abnormality associated with ASD is hyperserotoninemia, reported in 35–46% of ASD patients [37]. Serotonin is synthesized from the essential amino acid Trp. Chronic immune activation (such as intestinal inflammation) induces the activation of IDO1. When IDO1 is activated, it drives further Trp breakdown. However, the primary catabolism pathway for Trp is KP, which competes with the 5-HT synthesis pathway. A clinical study found that approximately 58.7% of ASD patients had IDO1 activation, which may mask hyperserotoninemia [38]. Even in the presence of intrinsic drivers of hyperserotoninemia, IDO1 activation suppresses serotonin levels within the normal range, thus masking underlying serotonin dysfunction. This also demonstrates the correlation between changes in Trp and 5-HT concentrations and gut microbiota dysbiosis.

Although IDO1 activation masks peripheral serotonin level abnormalities (because peripheral 5-HT cannot cross the blood–brain barrier), abnormal central 5-HT synthesis remains a core feature of ASD. Studies have found that children with ASD have abnormally elevated 5-HT synthesis capacity in their brains during the first few years of development, but this does not subsequently decline over time as in normal children. Further PET studies also revealed asymmetry in the synthetic capacity of the ASD brain hemispheres, which differs from that of typically developing children [39]. This effectively demonstrates that the abnormality of the 5-HT system in ASD is a dynamic, developmentally stage-specific dysregulation. Simultaneously, genetic and environmental factors also play a role. Animal model validation has shown that abnormal expression of the serotonin transporter gene (SLC6A4) can also lead to hyperserotoninemia and ASD-related behavioral abnormalities, but single genetic variants account for only a small percentage [40]. Another study reported that infants of mothers with a history of mental illness have a higher risk of being diagnosed with autism later in life, indicating a potential genetic risk for ASD [41]. Clinical studies have also shown that prenatal exposure to selective serotonin reuptake inhibitors (SSRIs) doubles the risk of developing ASD [42]. SSRIs are commonly used to treat depression and other mental illnesses and can antagonize serotonin transporters, leading to elevated serotonin levels in the brain. This interaction of developmental trajectory, genetic factors (such as SLC6A4 mutations), and environmental factors (such as prenatal SSRI exposure) collectively reduces serotonin transport efficiency, leading to disordered serotonin signaling and excessive accumulation of serotonin in the central nervous system. While hyperserotoninemia has been extensively studied as a potential biological endophenotype of ASD and exhibits partial heritability, the exact cause of hyperserotoninemia in ASD patients remains unclear.

### 4.3. The Mechanism by Which Gut Microbiota Affects ASD Through Trp Metabolism

**Figure 2:**
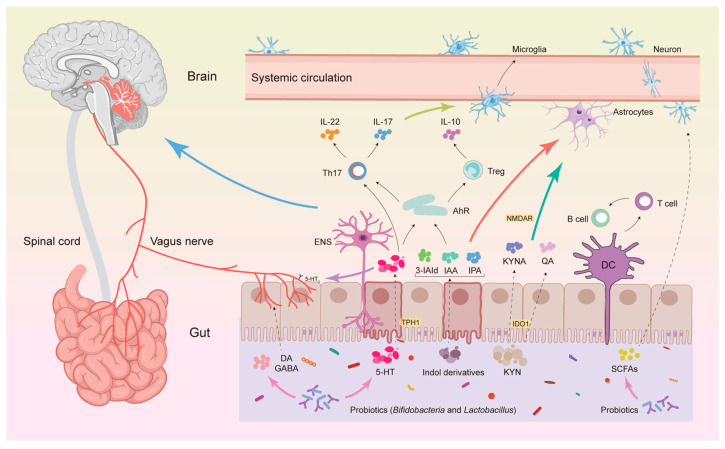
Trp metabolism–mediated gut–brain axis signaling pathway and its mechanism of action. This figure illustrates the complex bidirectional communication network between the gut and the brain, known as the gut–brain axis. It highlights two major communication pathways: the neural pathway, primarily mediated by the vagus nerve connecting the gut to the brainstem, and the humoral/systemic pathway, facilitated by circulatory factors in the bloodstream. Key interactions emphasized in the figure include: Gut-derived microbial metabolites (e.g., SCFAs), immune signaling molecules (e.g., IL-17), and Trp metabolites (e.g., indole derivatives, QA) can modulate peripheral immune cell activity and directly influence the status of central nervous system glial cells (microglia, astrocytes) and neurons. Simultaneously, serotonin produced from Trp metabolism and neurotransmitters such as DA and GABA can act directly on the vagus nerve, affecting brain signaling. These multifaceted interactions collectively regulate brain function. The balance between regulatory T cells (Tregs) and T-helper 17 (Th17) cells in the gut lamina propria is critical for maintaining immune homeostasis, and its disruption can also transmit signals to the brain. Additionally, the figure depicts the modulatory role of probiotics (specifically *Bifidobacteria* and *Lactobacillus*), which can influence this axis by regulating microbial composition, producing beneficial metabolites like SCFAs, and potentially influencing neurotransmitter levels. Conversely, the brain can modulate gut functions (e.g., secretion, motility, immune responses) via outputs from the autonomic nervous system, including the vagus nerve. This integrated perspective explains how gut health, particularly through the lens of Trp metabolism, profoundly influences brain function, providing important insights into ASD. Abbreviations: ENS, Enteric Nervous System; SCFAs, Short-Chain Fatty Acids; Treg, Regulatory T Cell; Th17, T-Helper 17 Cell; IL, Interleukin; DA, Dopamine; GABA, Gamma-Aminobutyric Acid; DC, Dendritic Cell.

The gut microbiota influences brain function and behavior by regulating Trp metabolism, and its mechanisms mainly revolve around three core pathways (Figure 2).

#### 4.3.1. The KP Pathway

95% of Trp is metabolized through the KP, which leads to several key metabolites through different branches, such as Kyn, KA, QA, pyridinecarboxylic acid (PA), NAD+, and adenosine triphosphate (ATP). Kyn can have harmful effects: it can induce symptoms of depression and anxiety, and has additional excitatory and neurotoxic effects. Subsequently, some Kyn is metabolized to KA, which has anti-anxiety and neuroprotective effects, while another portion is metabolized to QA, which has neurotoxic effects. The Kyn/Trp ratio reflects IDO1 activity, and the Kyn/KA ratio reflects its neurotoxic potential. Therefore, any changes in the regulation of the KP can lead to neurotoxic effects and have serious impacts on behavior and cognition [43]. A study by Bryn et al. found that the Kyn/KA ratio in children with ASD was significantly higher than in healthy controls, which may indirectly indicate an increased neurotoxic potential in children with ASD [44].

Mechanistically, under inflammatory conditions, the increase in pro-inflammatory factors leads to the activation of peripheral IDO1, thereby driving more Trp to be metabolized through the KP, producing neurotoxic QA. Kyn and QA are antagonists and agonists of the N-methyl-D-aspartate receptor (NMDAR), respectively. NMDAR is involved in the normal development of the central nervous system and can affect cognitive function and stereotyped behaviors [45]. Simultaneously, excessive accumulation of neurotoxic QA is closely related to oxidative stress and neuronal damage. QA can not only directly stimulate NMDAR receptors but also inhibit iron autoxidation by binding to iron ions, activating oxidative stress pathways and leading to increased lipid and protein peroxidation levels. This effectively disrupts neuronal function and activity, thereby affecting the brain and interfering with normal behavioral and cognitive expression [46]. The KP metabolites exert direct toxic effects on neurons by inducing redox imbalance, which is also one of the mechanisms by which they cause neuronal damage. An animal study found that NMDAR knockout mice could not establish normal social hierarchies, consistently playing a subordinate role among their littermates and exhibiting social withdrawal upon encountering unfamiliar mice. This suggests that incomplete development of NMDAR-dependent neurons impairs the ability to discern emotional states and leads to social regression [47]. Another study showed that gut microbiota transplantation from healthy subjects effectively improved depressive behavior induced by chronic restraint stress (CRS) in mice. *Roseidonella* played a key role, with its effective colonization significantly inhibiting enzymes responsible for Kyn and QA generation, thereby reducing neurotoxicity [48]. Furthermore, *Roseidonella* played a crucial role in protecting against CRS-induced synaptic loss, microglia activation, and astrocyte maintenance. Therefore, abnormal Trp metabolism can activate oxidative stress pathways by producing neurotoxic substances, leading to social and cognitive impairments. Targeted regulation of the gut microbiota provides a potential strategy for intervening in this pathological process.

#### 4.3.2. 5-HT Pathway

Elevated platelet 5-HT levels are one of the most stable biological markers of ASD [49]. Numerous studies have shown that the function of the 5-HT system in the cerebral cortex, subcortical regions, and brainstem of autistic patients is generally reduced, with an imbalance in 5-HT levels between the periphery and the central nervous system [50]. It is generally believed that during neurodevelopment, excessively high 5-HT levels in the brain may restrict neuronal growth through a negative feedback mechanism mediated by 5-HT 1A receptors, leading to the loss of 5-HT terminal neuron function and altering the development of target brain regions, thereby increasing the likelihood of ASD. This conclusion has also been validated by clinical trials [51]. A classic PET study found that in autistic children, serotonin synthesis gradually increases between the ages of 2 and 15, reaching 1.5 times the normal adult value, with no gender difference, effectively demonstrating the abnormal timeline of 5-HT development in the brains of ASD patients [52]. Another clinical controlled trial based on adult males showed no difference in 5-HT 1A receptor concentration between the two groups of subjects. A certain degree of imbalance exists in striatal density, and this receptor dysregulation is considered an adaptive change resulting from long-term dysfunction of the 5-HT system [53]. Simultaneously, 5-HT is a major target for medications treating anxiety and depression (such as SSRIs). Dysregulation of the 5-HT system in ASD patients makes them more prone to anxiety, depression, and obsessive compulsive symptoms, further illustrating a direct link between the system’s availability and core behavioral manifestations of autism [54].

HT plays a crucial role in intestinal motility, secretion, immune responses, and the neural regulation of the gut–brain axis. 90% of 5-HT originates from the gut. Due to the blood–brain barrier, gut-produced 5-HT has difficulty directly entering the brain, but it can indirectly affect the central nervous system through the gut–brain axis. 5-HT released by enterochromaffin cells in the gut can directly activate 5-HT3 receptors on vagal nerve afferent fibers, and then diffuse neural information about gut stimulation to the amygdala, locus coeruleus, and other areas of the brain via synapses or neurons in the tuberous ganglion [55,56]. Studies have found that ondansetron (a widely used 5-HT3 receptor antagonist) can effectively improve social interaction and behavioral inhibition in BTBR mice, exhibiting antidepressant effects and regulating 5-HT3 receptor function. It could serve as an effective strategy for simultaneously intervening in multiple core behavioral domains of ASD [57]. Common gastrointestinal symptoms in ASD patients (such as constipation, diarrhea, and abdominal pain) may also be related to abnormal intestinal 5-HT signaling. Excessively high 5-HT levels in the gut can activate specific receptors on intestinal epithelial cells (such as 5-HT2A and 5-HT7), leading to abnormal expression of tight junction proteins and causing “leaky gut” [58]. In this inflammatory state, the body releases large amounts of pro-inflammatory cytokines. Peripherally derived inflammatory signals activate microglia to produce more inflammatory factors locally in the brain, ultimately leading to depression, anxiety, and cognitive decline. Simultaneously, 5-HT can directly regulate the differentiation of immune cells (especially Th1 and Th17 cells), altering the local and systemic immune balance in the gut, and allowing pro-inflammatory cytokines to enter the circulation and affect the central nervous system. The gut microbiota directly or indirectly regulates the balance of 5-HT in the gut. A study of preschool children found that children with autism had a higher proportion of gastrointestinal symptoms and more severe symptoms compared to typically developing children. Specific gut bacteria and metabolites produced by the gut microbiota may play an important role in regulating the level of 5-HT secreted by intestinal cells [59]. For example, *Clostridium* species from the mouse and human microbiota can promote the biosynthesis of 5-HT in intestinal cells, significantly affecting host physiological functions and regulating gastrointestinal motility and platelet function [33]. In addition, short-chain fatty acids produced by some gut microbes from the consumption of carbohydrates in food have been shown to increase the level of Trp1 mRNA in intestinal cells, thereby promoting the production of 5-HT in the colon [60]. This regulation of 5-HT levels is an important pathway for the microbiota to maintain host physiological homeostasis. Another clinical trial found that in a lactic acid-rich environment, the abundance of *Akkermansia* was significantly increased, along with a rise in propionic acid concentration. This process significantly alters the expression of IDO1 and TPH1, shifting Trp metabolism from the KP to the 5-HT production pathway [61]. Simultaneously, the synergistic effect of lactic acid and gut microbiota enhanced the integrity of the intestinal barrier, thereby indirectly restoring the balance of the 5-HT system between the periphery and the brain. The gut microbiota is a crucial bridge connecting gastrointestinal symptoms and neuropsychiatric symptoms in autism patients, and 5-HT is an important intervention target within it.

#### 4.3.3. Indole and Its Metabolite Pathways

Unlike 5-HT, indole is entirely produced from Trp by gut microbes. Gut microbiota convert a very small amount of Trp into indole and its derivatives. In inflammatory states, this drives more Trp into the KP, leading to inhibition of the indole pathway. These indole compounds are important ligands for AhR. AhR activation is central to connecting the gut microbiota, intestinal barrier, immune system, and central nervous system. When AhR levels are reduced, on the one hand, the inability to effectively activate the AhR signaling pathway weakens the connections between intestinal epithelial cells, disrupting the integrity of the intestinal barrier; on the other hand, insufficient AhR activation disrupts gut immune homeostasis, leading to abnormal immune regulation. Simultaneously, the dysregulated immune system produces more pro-inflammatory factors, further exacerbating systemic inflammation and “leaky gut”. Previous studies have demonstrated several correlations between indole metabolites, brain activity areas (insula subregion, dmPFC/aMCC, IFGop, and S1), and patient symptoms (autism severity and alexithymia) [10]. Needham et al. found a significant negative correlation between fecal levels of several indoles (such as indole propionate, indole, n-formyl-o-aminobenzoic acid, and indole-3-carboxylic acid) and ASD severity [62]. Another clinical behavioral assessment study also showed that lower indole-lactate levels in ASD patients during aversion processing were significantly associated with increased activity in the right midinsula of the brain. The midinsula is the center for interoceptive, emotional, and chemoreceptive processing, further illustrating that indole metabolites can indirectly affect brain function, leading to the expression of some ASD-related behavioral symptoms [10].

A growing body of research reports that the Trp-AhR pathway demonstrates effectiveness in strengthening the intestinal mucosal barrier by regulating the expression and distribution of tight junction proteins. The proliferation of lactic acid bacteria in the gut allows for rapid adaptation to the rapidly changing intestinal environment [29]. They metabolize Trp into indole-3-methanol (I3M) and tryptophanamine. These two ligands can reduce pro-inflammatory cytokines induced by macrophage inflammatory markers, fatty acids, and lipopolysaccharide (LPS), and inhibit cell migration to chemokine sites, maintaining biodiversity and protecting the intestinal mucosa from damage. Simultaneously, ligand-activated AhR can promote the secretion of cytokines such as IL-22 and IL-17, regulate the release of antimicrobial peptides from intestinal epithelial cells, and improve the balance of the gut microbiota [63]. On the other hand, the indole derivative-AhR pathway can indirectly regulate central nervous system inflammation through the gut–brain axis. Some indole metabolites of Trp, such as IAA and IPA, can activate microglia. Microglia transmit signals through AhR in astrocytes. These two cell types communicate at the molecular level to mediate responses to central nervous system inflammation, thereby alleviating neuroinflammation. The two work together to create a more stable internal environment for the brain, effectively alleviating anxiety and mood swings, and thus improving the behavioral symptoms of ASD patients [64].

## 5. Probiotic Intervention: Targeting the Gut Microbiota–Trp Metabolic Axis

### 5.1. Probiotics Regulate Gut Microbiota

Probiotics have garnered significant attention for their health-promoting and disease-improving effects as adjunctive therapies. Probiotics can improve the gut microbiota through various mechanisms, including regulating gut microbiota structure and diversity, promoting mucus secretion to strengthen the intestinal barrier, and modulating immune responses and the indirect effects of their metabolites [65]. For example, *Lactobacillus rhamnosus* (LGG) is one of the most widely used and extensively studied probiotics, and its various biological characteristics demonstrate its potential application value [66]. Colonization of the probiotic LGG can competitively inhibit the growth of harmful bacteria (such as *Escherichia coli* and *Clostridium*), thereby slowing down changes in the gut microbiota. In vitro experiments showed that LGG and *Bifidobacterium lactis* Bb12 completely inhibited the growth of avian pathogenic *Escherichia coli* (APEC), a potential foodborne zoonotic pathogen, within 24 h. Subsequent animal experiments also demonstrated that oral administration of LGG competitively reduced APEC colonization in the chicken cecum, contributing to the maintenance of gut microbiota homeostasis [67]. Meanwhile, some antimicrobial compounds produced by probiotic metabolism, such as bacteriocins and organic acids, can exert beneficial effects through various mechanisms, including inhibiting or killing specific pathogens and regulating the intestinal environment to suppress pathogen growth [68,69,70].

Furthermore, probiotics have been shown to reduce intestinal permeability (“leaky gut”), maintain the integrity of the intestinal barrier, and, in terms of Trp metabolism, effectively prevent Trp leakage from the intestine, improving its utilization in the body. Simultaneously, probiotic use can indirectly inhibit the overactivation of IDO1 by reducing the release of pro-inflammatory factors (such as TNF-α and IL-6), allowing more Trp to be used in other metabolic pathways [71]. Probiotics can also regulate the host’s innate and adaptive immune responses, modulating the function of dendritic cells (DCs), monocytes/macrophages, and T and B lymphocytes, thereby enhancing the phagocytosis of invading intestinal pathogens, and thus regulating the intestinal flora and reducing intestinal inflammation [72]. It can be seen that probiotics have shown great therapeutic potential in inhibiting pathogens, promoting intestinal barrier repair, and regulating immunity.

### 5.2. Probiotics Regulate Trp Metabolism

Common probiotics fall into two main categories: Lactobacilli and Bifidobacteria. Additionally, based on their benefits to humans, some Gram-positive bacteria (such as *Lactococcus lactis* and *Streptococcus thermophilus*) and yeasts (such as *Saccharomyces boulardii*) are also classified as probiotics. In recent years, the regulatory effects of probiotics and their metabolites on the Trp metabolic pathway have become a research hotspot, especially in the gut–brain axis region. However, the results of interventions with different probiotic strains vary significantly, and the mechanisms of action of each strain are also different. Previous animal studies have shown that the KP in autistic rats is significantly disrupted and is closely related to the regulation of neurotransmitters and glial cells. Intervention with *Bifidobacterium longum* CCFM1077 significantly regulated QA levels in the brain, while reducing the activity of cerebellar microglia, effectively improving autistic behaviors in rat models such as repetitive stereotyped behaviors, learning and memory abilities, and despair, and regulating the KP metabolism in the peripheral system and brain [73]. Importantly, direct evidence that specific probiotic strains directly modulate the key downstream enzymes determining the Kyn/KA ratio (e.g., host KATs or KMO) remains limited in most studies; many reports infer KP rebalancing primarily from metabolite readouts and/or upstream IDO1-driven flux. In inflammatory settings, cytokine stimulation can regulate the expression of KP enzymes, providing a mechanistic basis for indirect probiotic effects on the neurotoxic branch [74]. In support of enzyme-level regulation at the expression level, a CRS mouse study reported that fecal microbiota transplantation from healthy donors improved depressive-like behavior and that targeted colonization with the beneficial commensal *Roseburia intestinalis* reduced Kyn and QA levels and altered host KP enzyme expression, including inhibition of IDO1 and 3-hydroxyanthranilate 3,4-dioxygenase (3HAO) and restoration of KAT2 expression [48]. Notably, KMO expression/activity was not directly quantified in this study; therefore, KMO-specific conclusions should be interpreted cautiously unless KMO is directly measured. Intervention with *Lactobacillus plantarum* DPUL-S164 can promote the production of indole-3-lactic acid (ILA) and the expression of tight junction proteins in the mouse gut, thereby activating the AhR signaling pathway and alleviating lipopolysaccharide-induced intestinal barrier damage and inflammation [75]. Xia et al. found that supplementation with *Lactobacillus plantarum* D266 can alter the abnormal composition of the gut microbiota, enhance tryptophan metabolism, and metabolize tryptophan into ILA, thereby activating AhR signaling and increasing IL-22 production to regulate intestinal homeostasis [76].

Numerous studies have also shown that probiotics can regulate Trp metabolism by increasing the abundance of beneficial bacteria in the gut, modulating neurotransmitters (such as glutamate, GABA, and DA), and increasing serotonin levels, thereby affecting the central nervous system and immune system [24]. *Lactobacillus plantarum* D-9 is a lactic acid bacterium that produces GABA [77]. It can alleviate depressive and anxious behaviors in mice under mild stress by increasing 5-HT concentration and regulating the imbalance of the hypothalamic-pituitary-adrenal axis. GABA is an inhibitory neurotransmitter in the central nervous system and a major target for treating anxiety disorders [78]. Its effects on various diseases are attributed to its regulation of multiple receptor functions through neural signal transmission and distribution in the central nervous system and peripheral organs. In another animal experiment, mice treated with *Lactobacillus rhamnosus* for a long period showed regional changes in central GABA receptors compared to control mice, indirectly regulating Trp levels and reducing stress-induced corticosterone levels and anxiety- and depression-related behaviors [79]. In another animal experiment using a dextran sulfate sodium (DSS)-induced mouse model, Huang et al. found that *Lactobacillus plantarum* DMDL9010 could inhibit neuroinflammation by upregulating 5-HT levels, reducing DSS-induced inflammatory responses, repairing intestinal barrier damage, and alleviating depressive behaviors in mice [80]. In addition to these indirect host-mediated effects, emerging in vitro evidence suggests some strains may contribute more directly to downstream KP products; for example, *Lactobacillus reuteri* was reported to preferentially synthesize kynurenic acid from kynurenine and release it extracellularly, supporting a potential direct microbial contribution to KA availability [81]. This demonstrates that specific probiotic strains can regulate Trp metabolism, thereby modulating neurotransmitters and immune responses, ultimately exerting beneficial effects on central nervous system function and behavior (such as ASD, anxiety, and depression), although the effects and mechanisms of different strains exhibit some specificity [82]. While animal experiments provide encouraging preclinical data, we must still remain cautious. The primary value of animal studies lies in offering crucial clues for understanding disease mechanisms and screening potential intervention targets. However, their efficacy and safety ultimately need to be validated in clinical trials and necessitating careful selection of probiotic strains for targeted intervention.

### 5.3. Evidence That Probiotics Mediate Trp Metabolism to Improve Symptoms of ASD

Probiotics have been explored as a strategy to modulate ASD-related symptoms via the microbiota–gut–brain axis [83]. In the context of this Trp-focused review, the proposed sequence is: (i) probiotics reshape gut microbial community structure and functional capacity, (ii) this alters gut-derived neuroactive signaling and neurotransmitter-related pathways (notably serotonergic and GABAergic-related processes, among others), and (iii) these upstream changes can re-balance host Trp metabolism across 5-HT, Kyn, and microbial indole/AhR-linked routes, thereby influencing immune tone, neuroinflammation, and brain function. Accordingly, clinical interpretation should distinguish changes in gastrointestinal (GI) and associated symptom domains from changes in core ASD symptoms, and should consider whether Trp-pathway engagement is directly quantified in a given trial.

#### 5.3.1. Overall Pattern

Across published studies, probiotic interventions show heterogeneous outcomes in children with ASD. A recurring pattern is that GI symptoms and associated behavioral domains (e.g., sleep, irritability, anxiety, attention) improve more consistently than clinician-rated core ASD symptoms. This distinction is clinically important because a reduction in GI burden may yield meaningful functional benefits, yet it should not be conflated with robust modification of core ASD symptom domains.

However, minimal clinically important difference (MCID) thresholds are not uniformly established for many ASD outcomes (e.g., ABC, SRS, ADOS), and GI-specific MCIDs have rarely been validated in ASD populations. Where available, anchor- or distribution-based benchmarks can provide context; for example, changes of approximately 2–3.75 points on the Vineland-II Composite score have been proposed as an MCID in ASD. In many ASD trials, clinical “response” is therefore operationalized using anchor-based criteria (e.g., ≥25% reduction on ABC-I/SRS together with a favorable CGI-I rating), rather than universally accepted MCID thresholds [84,85,86].

#### 5.3.2. Trials with Improvement

Several studies report improvements in symptom scales and/or GI indices. Shaaban et al. evaluated a multi-strain probiotic formulation (*Lactobacillus acidophilus*, *L. rhamnosus*, and *Bifidobacterium longum*) and reported improvement in behavioral measures and GI symptoms [87]. Guidetti et al. conducted a randomized, double-blind crossover trial and observed improvements in some behavioral/adaptive domains, parental stress, and GI symptoms in subsets of participants [88]. Lin et al. reported that *Bifidobacterium breve* BF839 improved behavioral scales and GI symptoms over 16 weeks, accompanied by microbiota shifts [89]. Khanna et al. (single-blind RCT) reported improvements in ASD-related behaviors (SRS-2/ABC-2) and GI symptom scores over 3 months without major safety concerns [90]. Open-label or combined-intervention studies also reported improvements in ASD severity and GI symptoms together with metabolic shifts related to neurotransmission (e.g., glutamate/GABA- and serotonergic-related signals) [91].

Because this review focuses on Trp metabolism, it is important to separate studies that infer Trp-pathway involvement from those that directly quantify Trp-related metabolites in humans. Among the relatively few probiotic trials in ASD that measured peripheral Trp-pathway readouts, Wang et al. reported a baseline profile consistent with altered serotonergic and the KP metabolism in ASD (including elevated peripheral 5-HT and 5-HIAA and reduced Kyn relative to controls) and showed that a multi-strain probiotic plus fructo-oligosaccharide intervention shifted these indices toward a control-like direction. Specifically, after intervention, the probiotic + FOS group exhibited significant reductions in 5-HT and 5-HIAA together with an increase in kynurenine, whereas the placebo group did not show comparable changes. The authors interpreted these coordinated shifts as a partial recovery of Trp–the KP balance and an alleviation of a hyper-serotonergic peripheral state in ASD. They further reported correlations between multiple bacterial taxa and Trp-related metabolites and supported pathway-level interpretation using predictive functional profiling (e.g., KEGG/PICRUSt-based predictions) [92]. Notably, these findings provide rare clinical support for Trp-pathway engagement; however, the evidence is based primarily on peripheral metabolite measurements and association analyses, so mechanistic interpretations should be viewed as supportive rather than definitive without standardized longitudinal multi-omics and predefined responder analyses.

#### 5.3.3. Trials with Mixed or Null Results

Not all trials demonstrate significant improvement in core ASD symptoms. Liu et al. reported that *Lactobacillus plantarum* PS128 improved certain secondary outcomes but did not show clear improvement in core symptoms; GI follow-up was incompletely captured and microbiome data were not available, complicating mechanistic attribution [93]. Kong et al. suggested potential synergy when PS128 was combined with intranasal oxytocin, implying that probiotics may function better as adjuncts than as standalone treatments in some contexts [94]. Accordingly, probiotics should currently be interpreted primarily as adjunctive interventions—particularly for children with prominent GI comorbidities—until larger trials establish standalone efficacy on predefined core endpoints. Arnold et al. performed a placebo-controlled crossover pilot in children with persistent GI symptoms and reported trends in GI-related quality-of-life outcomes, but limited statistical power constrained definitive conclusions [95]. A key nuanced example is the 6-month double-blind RCT of the De Simone Formulation (DSF), which did not show significant clinician-rated core symptom change in the whole sample but reported signals in specific subgroups [96]. Neurophysiological data can partly reconcile “clinical null” findings: Billeci et al. reported that DSF supplementation shifted resting-state EEG patterns toward a more typical profile while behavioral changes were modest, supporting biological engagement that may not immediately translate into detectable core symptom change [97]. Similarly, other work indicates that probiotics can alter microbiome diversity/composition without consistent robust behavioral benefit across the entire ASD sample, reinforcing the need for stratified interpretation [98].

#### 5.3.4. Baseline and Responders

Baseline gut microbiome heterogeneity is likely an important determinant of probiotic efficacy in ASD, as initial community structure can influence colonization resistance, niche availability, and downstream metabolic outputs, including tryptophan-pathway metabolites. Human evidence suggests that probiotic colonization and host responsiveness are highly individualized, supporting stratified or responder-based trial designs [99]. Baseline microbiome variation in ASD is closely intertwined with dietary structure, and microbiome differences reported in ASD may be substantially mediated by ASD-related dietary preferences (e.g., restricted/repetitive behaviors associated with low-diversity diets). This diet-mediated perspective highlights a “behavior → diet → microbiome” pathway that can complicate causal interpretation. Consistent with this framework, Yap et al. conducted a large stool metagenomics analysis and reported limited direct associations between ASD diagnosis and overall microbiome profiles, with *Romboutsia timonensis* being the only taxon associated with diagnosis under their significance criteria. In contrast, the study identified robust associations between microbiome variation and non-diagnostic baseline factors—particularly dietary patterns and stool consistency—supporting the view that diet-related and GI-context variables may drive a substantial proportion of the apparent ASD–microbiome signal [100].

Their mediation analyses suggested that ASD-related restricted interests were linked to a less diverse diet, which was associated with reduced microbial alpha-diversity and altered stool consistency—consistent with a “behavior → diet → gut physiology/microbiome” pathway that may dominate apparent ASD–microbiome associations. Consistently, integrative studies combining dietary assessment and metagenomics have identified ASD-specific diet–microbiome association networks, where poorer dietary quality correlates with aggregated core symptoms, GI comorbidities, and atypical eating behaviors [101]. Beyond diet, chronic stress-related physiology may also feed back on gut microbial ecology via neuroendocrine and immune routes, reinforcing bidirectionality in the microbiota–gut–brain axis [102].

Collectively, these findings suggest that “responders” may be better defined by a composite of baseline microbiome configuration, dietary patterns, GI phenotype, and behavioral context rather than diagnosis alone. Accordingly, future ASD RCTs would benefit from comprehensive baseline profiling (microbiome plus targeted Trp-related metabolomics), standardized dietary assessments, and pre-specified responder definitions to strengthen mechanistic interpretability and clinical translation.

#### 5.3.5. Sources of Heterogeneity

The apparent contradictions across studies likely reflect multiple interacting sources of heterogeneity:

(1) Outcome selection and measurement sensitivity: clinician-rated core measures (e.g., ADOS-based tools) may be less sensitive to short-term change than caregiver-rated questionnaires, while caregiver measures can be more susceptible to expectancy/placebo effects.
(2) Baseline phenotype and stratification: ASD is heterogeneous; GI symptom burden, age/developmental stage, baseline severity, and dietary selectivity can influence response and should be pre-specified for subgroup analyses.
(3) Strain/formulation specificity: single-strain vs. multi-strain products, synbiotics (probiotic + prebiotic), and product viability differ markedly, limiting cross-study comparability.
(4) Dose–duration heterogeneity: trials vary by orders of magnitude in CFU/day and by intervention duration (weeks–months), precluding simple dose–response inference without harmonized reporting.
(5) Concomitant interventions and diet: ongoing behavioral therapies, medications, dietary modifications, or adjunctive agents (e.g., oxytocin) can confound attribution of effects to probiotics alone.
(6) Limited pathway-level measurement: many trials do not quantify Trp-pathway metabolites (e.g., Kyn/KA-related readouts), making it difficult to connect clinical outcomes to the Trp-centered mechanistic framework.

Together, these factors argue for future RCTs that (i) report strains and CFU/day transparently, (ii) standardize and pre-specify core vs. associated endpoints, and (iii) incorporate baseline stool profiling with targeted Trp-pathway metabolomics to enable responder analyses and mechanistic interpretation (Table 2).

#### 5.3.6. Dose–Response, Duration–Response, and Formulation Considerations

A formal dose–response and duration–response synthesis remains challenging because most ASD probiotic trials employ single-dose designs, CFU/day spans several orders of magnitude, and reporting of viable counts and adherence is inconsistent, which precludes robust modeling of strain-specific optimal dosing ranges (10^6^–10^11^). Importantly, higher CFU should not be assumed to confer greater clinical benefit: authoritative guidance notes that products with higher CFU counts are not necessarily more effective than those with lower CFU counts, and human dose-ranging evidence indicates that increasing probiotic dose can alter gastrointestinal tolerance (e.g., significantly looser stool consistency with higher dosing). Consistent with a non-linear, responder- and endpoint-dependent framework, cross-trial comparisons in ASD do not support a simple “more is better” interpretation; for example, subgroup/domain-specific signals have been reported with relatively low-dose single-strain interventions (e.g., BF839 for 16 weeks) [84], whereas higher-dose regimens do not consistently translate into clinician-rated core symptom improvement (e.g., PS128 for 4 months; DSF over 6 months with overall null core effects but subgroup signals). Duration appears relevant but not sufficient: improvements are more commonly reported for GI and associated domains with ≥8–12-week exposure, yet longer duration does not guarantee overall core symptom change [89,92,95,97,103]. With respect to formulation, direct head-to-head comparisons between single-strain and multi-strain products in ASD remain rare; therefore, superiority cannot be concluded. In the broader probiotic literature, efficacy is highly strain- and disease-specific and multi-strain mixtures are not uniformly superior to single strains, while methodological recommendations emphasize analyzing single- vs. multi-strain interventions separately and reporting per-strain dose and viability transparently [104,105]. Mechanistically, inter-strain antagonism within mixtures has also been demonstrated in vitro, providing a plausible basis for why adding strains may not yield additive benefits [106]. Overall, future ASD RCTs should incorporate dose-ranging arms, harmonized follow-up windows, explicit CFU/day (and viability) reporting, and pre-specified tolerability and responder analyses to enable clinically interpretable dose– and duration–response inference.

### 5.4. Clinical Positioning of Probiotics in ASD Management

Dietary interventions are frequently used in ASD management and include elimination diets (e.g., gluten-free/casein-free, GFCF), ketogenic or modified ketogenic approaches, and broader anti-inflammatory or low-additive dietary strategies. For example, the ScanBrit randomized controlled single-blind study evaluated a GFCF diet in children with ASD and reported evidence suggestive of developmental/behavioral improvements in subsets of participants, while also noting that non-dietary factors could not be fully excluded as contributors in the absence of a placebo condition [7]. In addition, a pilot interventional study of a modified ketogenic diet in children with ASD reported microbiome shifts alongside changes in inflammatory cytokines and circulating brain-related miRNAs after 4 months of intervention, supporting biological engagement but still requiring larger controlled trials for definitive clinical conclusions [107]. More recently, a randomized nutritional intervention trial compared a structured anti-inflammatory diet (NeuroGutPlus) with multi-strain probiotic supplementation and no intervention over 12 weeks; the anti-inflammatory diet produced broader and more consistent immunoregulatory changes (e.g., reduced IFN-γ and stabilizing immune profiles), whereas probiotics induced a distinct immune signature, highlighting that dietary strategies can exert systemic effects that may differ qualitatively from probiotics alone [108].

Crucially, the clinical interpretation of any microbiota-targeting strategy must consider the complexity of diet in ASD: restricted/repetitive eating patterns, selective food avoidance, and variability in macronutrient/fiber intake can all reshape the gut microbiome and GI physiology, thereby confounding attribution of changes to probiotics alone. Accordingly, combined or multimodal approaches are increasingly explored. As one example, a precision synbiotic (probiotic + prebiotic) open-label study reported increased alpha diversity and improvements in GI discomfort together with signals in ASD-related symptom domains, supporting the feasibility of combining microbial supplementation with dietary substrate support, while also underscoring the need for randomized, placebo-controlled confirmation [109]. Finally, direct head-to-head comparisons between probiotics (as standalone interventions) and established pharmacological treatments in ASD remain limited; moreover, commonly used medications such as risperidone and aripiprazole are indicated for irritability associated with autistic disorder rather than core symptoms, which further complicates direct efficacy comparisons across target domains [110,111]. Therefore, current evidence supports positioning probiotics primarily as adjunctive or domain-targeted strategies (particularly for GI and associated symptom domains) rather than substitutes for established clinical management.

## 6. Challenges and Future of Probiotic Intervention for ASD

### 6.1. Current Challenges

The therapeutic potential of probiotics has been extensively studied in various diseases, demonstrating significant value in both clinical treatment and commercial applications. However, the steady expansion of the probiotic market has brought more uncertainties to the evaluation of clinical efficacy and safety, primarily due to safety issues arising from strain specificity and individual differences. Probiotics exhibit significant strain specificity; even different strains of the same genus and species show differences in Trp metabolism or immune regulation. Furthermore, various factors directly or indirectly contribute to individual differences, including the host’s initial gut microbiota state, diet, and lifestyle [112]. This is particularly true for children with ASD whose immune systems may be abnormal, where probiotic intervention periods are often short-lived. Coupled with the uncertainty of the colonization process, long-term safety data are still lacking. Additionally, most studies remain at the observational stage of clinical symptoms, and the molecular mechanisms by which probiotics precisely target specific metabolic pathways and neural circuits remain unclear.

### 6.2. Future Directions

Future research directions for probiotics mainly focus on achieving personalized treatment. This involves combining multi-omics technologies to accurately assess host microecological characteristics, enabling targeted probiotic interventions to improve the body’s condition [113]. Firstly, existing research has utilized gene editing to modify probiotics, developing “next-generation probiotics” with specific characteristics, including improved intestinal survival, precise targeting of specific gastrointestinal regions, and the ability to produce therapeutic compounds [114]. In addition to modifying natural strains, precise gene editing can also design “engineered probiotics” that can specifically secrete neuroactive substances (such as ILA and GABA) or degrade neurotoxins. Several challenges and ethical issues remain to be addressed before these interventions are widely accepted clinically. The gut microbiome is a highly dynamic and complex ecosystem. Introducing new probiotic strains—especially genetically modified ones—can disrupt the microbial balance, leading to unforeseen health problems such as dysbiosis, opportunistic pathogen overgrowth, or metabolic disorders. Furthermore, engineered probiotics often fall between the categories of food, supplements, and pharmaceuticals, lacking standardized testing methods, resulting in regulatory uncertainty. Therefore, clinical trials involving engineered probiotics must fully disclose potential risks and long-term effects. Patients must have autonomy to accept or reject microbiome interventions based on informed decision-making, and strict microbiome-related health data privacy regulations must be implemented.

Secondly, given the complexity of gastrointestinal problems in ASD patients, a crucial future research direction is developing personalized combination therapies. The synergistic effects of combining synbiotics (probiotics and prebiotics), specific dietary interventions, or conventional behavioral therapies can achieve synergistic effects from multiple levels, including gut microbiota and behavioral psychology. On the other hand, identifying biomarkers that can predict probiotic treatment responses remains key to probiotic therapy research in ASD. Utilizing multi-omics technologies (metagenomics, metabolomics, etc.) to find predictive biomarkers is a core element in realizing personalized medicine. Future research should focus on achieving personalized applications of probiotics through precise targeting guided by multi-omics and multi-modal synergistic intervention.

## Figures and Tables

**Table 1:** Key characteristics of the three major metabolic pathways of Trp.

Metabolic Pathway	Primary Sites	Key Enzymes	Major Metabolites	Main Functions/Effects	Related Diseases or Physiological Processes
Kynurenine pathway (KP)	Liver, immune cells, epithelial cells	TDO, IDO1, IDO2	Kynurenine (Kyn), kynurenic acid (KYNA), quinolinic acid (QA), NAD+	Immune regulation, energy metabolism, neuroregulation	Alzheimer’s disease, depression, schizophrenia, etc.
Serotonin pathway (5-HT)	Intestinal enterochromaffin cells, raphe nuclei neurons	TPH1, TPH2	Serotonin (5-hydroxytryptamine, 5-HT)	Regulates gut motility and secretion, vasodilation, neurotransmission, mood and cognition	Mood disorders, irritable bowel syndrome, etc.
Indole pathway	Gut microbiota	Bacterial tryptophanase and related enzymes	Indole, indole acrylic acid, indole-3-acetic acid (IAA), indole-3-propionic acid (IPA), etc.	Activates AhR, promotes IL-6/IL-17/IL-22, maintains intestinal barrier and homeostasis	Inflammatory bowel disease, metabolic syndrome, etc.

**Table 2:** Clinical trials of probiotic interventions in children with ASD.

Sample Size	Intervention/Strain (s)	CFU	Duration	Outcome Measures	Main Findings (Behavior/Symptom Improvement)	Citation
ASD children (n = 30); healthy controls (n = 30)	Multi-strain probiotics: *Lactobacillus acidophilus*, *L. rhamnosus*, *Bifidobacterium longum*	1 × 10^8^	3 months	Behavioral scales (ATEC, etc.), GI six indices	Significant improvement in disruptive behavior, antisocial behavior, anxiety, and communication deficits	Shaaban et al., 2018 [87]
ASD children (n = 61)	Multi-strain probiotics: *L. fermentum* LF10, *L. salivarius* LS03, *L. plantarum* LP01, *B. longum* DLBL07–11	1 × 10^10^	8 months total (each intervention period 3 months + washout)	GI Severity Index, PSI, VABS, ASRS	In a subset of ASD participants, behavioral severity decreased; communication/adaptive behavior and parent stress improved; GI symptoms generally improved	Guidetti et al., 2022 [88]
ASD children (n = 60, 2–10 years)	*Bacteroides fragilis* BF839	1 × 10^6^	16 weeks	Behavioral scales and GI symptom assessments, ABC, CARS, SRS, GSRS, etc.	Improved behavior and GI symptoms in ASD children (overall and in some subgroups significant)	Lin et al., 2024 [89]
ASD children (n = 180)	Powder formulation containing 12 probiotic strains	9 × 10^9^	3 months	SRS-2, ABC-2, GSI	Probiotic treatment significantly improved ASD-related behaviors and gastrointestinal symptoms, with behavioral gains paralleling GSI improvement.	Khanna et al., 2025 [90]
ASD children (n = 53, 3–12 years)	*Bifidobacterium animalis* subsp. *lactis* Probio-M8 + moderate-carbohydrate diet	1 × 10^11^	12 weeks	CARS, GSRS	Significant improvements in ASD and GI symptoms; modulation of glutamate/GABA/5-HT-related metabolism	Li et al., 2024 [91]
ASD children (n = 26)	Multi-strain probiotics + FOS: *B. infantis* Bi-26, *L. rhamnosus* HN001, *B. lactis* BL-04, *L. paracasei* LPC-37	1 × 10^10^	Up to 108 days (assessed at days 0/30/60/108)	ATEC, GI indices; SCFAs; neurotransmitters/metabolites incl. 5-HT/HVA	Reduced ASD and GI symptoms; SCFAs increased; hyper-serotonergic state alleviated; some putative pathobionts decreased	Wang et al., 2020 [92]
ASD children (82 randomized; 86 assessed; 2.5–7 years)	*Lactobacillus plantarum* PS128	3 × 10^10^	4 months	Attention, hyperactivity, impulsivity, oppositional defiant behaviors	Secondary outcomes improved; no significant improvement in core symptoms	Liu et al., 2023 [93]
35 participants	Single-strain *Lactobacillus plantarum* PS128; from week 16 onward, both groups additionally received intranasal OXT	6 × 10^10^	28 weeks	SRS, ABC, CGI-I, GI (GSI), inflammatory markers, fecal microbiota	PS128 combined with oxytocin produced greater social and behavioral improvements than oxytocin alone, suggesting a synergistic effect.	Kong et al., 2021 [94]
ASD children (n = 13, 3–12 years)	Multi-strain probiotics (VSL# 3/Visbiome)	9 × 10^11^	8 weeks	Behavioral and GI-related scales	Improvements vs. baseline but not statistically significant vs. placebo (small sample size)	Arnold et al., 2019 [95]
ASD children (n = 85)	Multi-strain probiotic DSF (De Simone Formulation)	4.5 × 10^11^	6 months	ADOS-2, etc.	Overall no significant improvement in core symptoms	Santocchi et al., 2020 [96]
ASD children (n = 46, EEG subset)	Multistrain Vivomixx^®^ (*S. thermophilus*, *B. breve*, *B. longum*, *B. infantis*, *L. acidophilus*, *L. plantarum*, *L. paracasei*, *L. delbrueckii*)	No specific explanation	6 months	ADOS-2, CARS, SCQ, RBS-R, CBCL, VABS-II, GSI, and resting-state EEG (power, coherence, asymmetry)	Resting-state EEG shifted toward a more typical activity pattern, while behavioral changes were only modest.	Billeci et al., 2023 [97]
ADHD children (n =39) ASD children (n = 41)	Multistrain probiotic preparation	1 × 10^9^	12 weeks	SRS-2, CBCL, BRIEF-2, SDSC, CPT/K-CPT2, fecal 16S rRNA microbiome	Probiotic intervention markedly altered gut microbiota, including increased alpha diversity in ASD, but yielded no consistent robust behavioral benefit.	Novau-Ferré et al., 2025 [98]

## Data Availability

No new data were created or analyzed in this study. Data sharing is not applicable to this article.
